# Cold atmospheric plasma as antiviral therapy – effect on human herpes simplex virus type 1

**DOI:** 10.1099/jgv.0.001382

**Published:** 2020-01-21

**Authors:** Oskar Bunz, Kemal Mese, Christina Funk, Maximilan Wulf, Susanne M. Bailer, Andree Piwowarczyk, Anja Ehrhardt

**Affiliations:** ^1^​ Institute of Immunology, Centre for Biomedical Education and Research (ZBAF), School of Medicine, Faculty of Health, Witten/Herdecke University, Witten, Germany; ^2^​ Department of Prosthodontics, School of Dentistry, Faculty of Health, Witten/Herdecke University, Witten, Germany; ^3^​ Institute of Virology and Microbiology, Centre for Biomedical Education and Research (ZBAF), School of Human Medicine, Faculty of Health, Witten/Herdecke University, Witten, Germany; ^4^​ Fraunhofer Institute for Interfacial Engineering and Biotechnology, Stuttgart, Germany; ^5^​ Institute for Interfacial Engineering and Plasma Technology IGVP, University of Stuttgart, Stuttgart, Germany

**Keywords:** cold atmospheric plasma, CAP, herpes Simplex, HSV-1, antiviral

## Abstract

In previous studies, cold atmospheric plasma (CAP) was explored as an antibacterial and antiviral agent for the treatment of chronic wounds. The aim of the present study was to investigate whether CAP may also be suitable as an antiviral therapy against herpes simplex virus type 1 (HSV-1). HSV-1 most frequently manifests as recurrent herpes labialis, but it can also cause encephalitis, conjunctivitis or herpes neonatorum as a perinatal infection. HSV-1 encoding the reporter gene GFP was propagated. The CAP dose for HSV-1 treatment was gradually increased, ranging from 0–150 s, and aciclovir was used as a positive control. After CAP treatment, the virus suspension was applied to a standard HSV research cell line (Vero cells) and the neuroblastoma cell line SH-SY5Y as a model for neuronal infection. The results showed that plasma treatment had a negligible antiviral effect on HSV-1 in both Vero- and SH-SY5Y cells at high dose. However, when we lowered the viral load 100-fold, we observed a significantly decreased number of internalized HSV-1 genomes 3 h post-infection for CAP-treated viruses. This difference was less pronounced with respect to GFP expression levels 24 h post-infection, which was in sharp contrast to the acyclovir-treated positive control, for which the viral load was reduced from 95 to 25%. In summary, we observed a low but measurable antiviral effect of CAP on HSV-1.

## Introduction

Herpesviruses have a unique four-layer structure. The nucleus contains the large, double-stranded DNA genome and is encapsidated by an isosapentahedral capsid consisting of capsomers. The capsid is surrounded by an amorphous protein layer, which is the tegument. The outermost envelope consists of a glycoprotein-containing lipid double-layer envelope [1]. There are more than 100 known herpesviruses, but only 9 of them infect humans. These viruses are divided into three groups, α-, β- and γ-herpesviruses. Herpes simplex virus type 1 and 2 (HSV-1 and HSV-2) belong to the α-herpesviruses. HSV-1 and HSV-2 only have ~40 % genomic homology [2], but they are very similar in their clinical symptoms [3]. Mucocutaneous manifestations of herpes simplex virus infections include gingivostomatitis [1], aphtous ulcerations [4], genital herpes [3, 5] and herpetic keratitis [6]. HSV-1 infections most frequently manifest as recurrent herpes labialis. Moreover, HSV-1 can cause encephalitis, conjunctivitis or herpes neonatorum as a perinatal infection.

Herpes simplex viruses first replicate in epithelial cells and produce characteristic vesicles. They then move along the sensory nerves to the dorsal root ganglia [7], where latency is established after an initial replication phase. The reactivation of the virus after latency is not yet fully understood [8, 9]. Following reactivation, the virus spreads back from the ganglion to initiate new skin or mucosal lesions.

Therapy of HSV-1 and HSV-2 infections depend strongly on the clinical localization. Aciclovir, valaciclovir or famciclovir are most commonly used topically, orally or intravenously [10–12].

Most antiviral agents are DNA/RNA polymerase inhibitors. A prominent type is aciclovir, which represents the first selective antiviral agent. Since its discovery in 1977 [13, 14], aciclovir has been used as a standard agent to treat HSV-1 infections. Aciclovir is a prodrug, which is activated by conversion to its triphosphated form. This reaction is mediated by the viral thymidine kinase and only occurs in infected cells. The activated aciclovir acts as a structural analogue to guanosine triphosphate. HSV-1 resistance to aciclovir has been reported recently [15, 16]. About 95 % of the described aciclovir resistance is based on mutations in the viral thymidine kinase gene or the DNA polymerase gene [15, 17]. As a result, researchers are constantly searching for new therapies to prevent the development of resistance.

One alternative therapeutic option could be cold atmospheric plasma. Plasma can also be called the fourth aggregate state and has properties that differ from those of the other states (solid, liquid, gas). If further energy is added to the gaseous state, emissions are produced that consist of visible light, ultraviolet (UV) radiation, electromagnetic waves and additionally ions and electrons, free radicals and other reactive species, such as reactive oxygen and nitrogen species (RONS). After reaction with water, these reactive species can induce hydrogen peroxide.

Cold atmospheric plasma (CAP) is a rather new technology, which is mainly used in dermatology and wound healing. Gases such as argon, helium or oxygen are partially ionized and energized by various methods. The partially ionized gases only lead to temperatures below 40 °C [18], which makes them attractive for medical applications. An increasing number of studies have demonstrated that CAP may be suitable for the treatment of chronic wounds [19–21]. This effect is mainly attributed to its bacteriostatic and bactericidal [22–25] effect, but also to the stimulated proliferation of basal epidermal keratinocytes [26]. UV light, reactive oxygen and nitrogen species have been described as the mechanisms of action [21]. These properties make CAP attractive for applications in the fields of medicine, healthcare, food processing and air pollution control.

Due to the non-specific effect of induced hydrogen peroxide and UV light, CAP could also have an antiviral effect. So far, the focus of research on antiviral effects of CAP seems to have been on the treatment of food for the prevention of transmissible viral diseases. For example, the effect of the argon-based gas plasma beams on norovirus was investigated in an *in vitro* study [27]. Moreover, plasma-activated water was successfully used to sanitize water containing bacteriophages [28]. In a prospective randomized placebo-controlled study CAP showed a significant reduction of pain and led to a more rapid clinical improvement of herpes zoster [29]. In addition, herpesviruses have already been examined *in vitro* and a reduction of HSV-1 infection of corneal epithelial cells was demonstrated [30]. Our previous study showed the different effects of CAP on various adenovirus types. CAP had an antiviral effect on certain adenovirus types, while no reaction or increased virus uptake was observed for other adenovirus types [31]. In the present study, the antiviral effect of CAP on HSV-1 was investigated utilizing different cell lines.

## Methods

### Cell culture

Vero cells and SH-S5SY cells were cultured under a humidified atmosphere of 5 % CO_2_ and 37 °C in a tissue culture dish until confluency was reached. The nutrient medium consisted of Dulbecco’s Modified Eagle’s Medium (PAN-Biotech GmbH, Aidenbach, Germany) supplemented with 10 % foetal bovine serum (FBS) (PAN-Biotech GmbH, Aidenbach, Germany) and 1 % penicillin/streptomycin (PAN-Biotech GmbH, Aidenbach, Germany). The cells were counted and seeded at a density of 1×10^5^ per well in 24-well tissue culture plates in triplicate for each test group.

### BAC mutagenesis

For HSV-1 virus production, a bacterial artificial chromosome (BAC) encoding the reporter gene GFP [pHSV1(17^+^)LoxGFP] was used. This BAC was generated using pHSV1(17^+^)Lox and the FRT/Flp recombination system in *Escherichia coli* DH10B. First, a resistance cassette flanked by mFRT sites was introduced into the viral genome between two tail-to-tail-oriented genes. The resistance cassette was amplified using PCR and oligos with 50 bp homologous regions flanking the insertion region. Insertion was accomplished using homologous recombination via the Red recombination system of phage λ encoded on plasmid pKD46 [32]. After verification of the correct insertion by restriction pattern analysis and local DNA sequencing, the resistance cassette was removed from the viral genome through the expression of plasmid-encoded recombinase Flpe, leaving one mFRT site behind. Finally, the BAC DNA was again verified before a transgene encoding GFP under the control of CMV promoter was integrated via Flpe-mediated recombination.

### Virus production

For HSV-1 virus [HSV1(17+)Lox] production, a bacterial artificial chromosome (BAC) was transfected into Vero cells and reconstituted virus was propagated in the same cell line. For transfection, Lipofectamin 2000 (Thermo Fisher Scientific, Waltham, MA, USA) was applied using a standard protocol. One six-well plate containing 3×10^5^ Vero cells was transfected with 1.6 µg BAC DNA and cultured until a cytopathic effect (CPE) was visible. Subsequently, 1 ml supernatant was transferred to a 25 cm^2^ flask and cultured until CPE was visible. This latter step was repeated one more time and after CPE was observed, supernatant and cells were harvested and frozen in a falcon tube at −80 °C. To release more virus particles, the suspension was thawed and centrifuged (1000 ***g***, 10 min, 4 °C) to separate cell debris. One millilitre aliquots were filled into cryotubes and stored as a stock at −80 °C.

### Plasma treatment

The treatment of HSV-1 with CAP (kINPen med, neoplas GmbH, Greifswald, Germany) was conducted by treating the virus medium stock. Three millilitres of virus medium suspension in a six-well plate were treated by different CAP time dosages (no treatment, 30 s, 60 s, 90 s, 120 s, 150 s). A distance of 15 mm to the medium surface was maintained. The CAP engine was operated using argon gas (purity 4.8) with the entry pressure set to 2.5 bar and the flow rate set to 5 l min^−1^.

### Aciclovir as a positive control for antiviral treatment

Aciclovir (aciclovir-ratiopharm, ratiopharm GmbH, Ulm, Germany) was used as a control to compare the effect of CAP with the standard medication. A concentration gradient between 0 and 3 µg ml^−1^ aciclovir was evaluated. Aciclovir was mixed with the virus suspension and added to the cells.

### Infection of cells

The infectivity of the virus suspension was investigated using a concentration gradient (0 to 60 µl HSV-1 suspension). A dose of 40 µl of the HSV-1 suspension on 1.5×10^5^ Vero cells resulted in ~90 % GFP-positive cells as measured by flow cytometry. Thus, a dose of 40 µl was applied for the following experiments. One hundred and 20 microlitres of the virus suspension was added to 3 ml medium and transferred to the Vero cells after the above-mentioned CAP treatment time doses. The infected and treated medium was divided into triplicates (1000 µl medium per 24-well plate). After infection, the cells were cultured for 24 h under a humidified atmosphere of 5 % CO_2_ and 37 °C.

### Evaluation of HSV-1 transduction efficiency after CAP treatment

The transduction efficiencies of tagged HSV-1 in Vero cells after treatment with CAP were measured by determining the reporter gene (GFP) expression levels. Twenty-four hours after infection, flow cytometry analyses (Navios Flow Cytometer, Beckmann Coulter, Krefeld, Germany) were performed. The percentage of GFP-positive cells and the mean fluorescence intensity in arbitrary units (AU) were evaluated. All experiments were performed in triplicate and repeated three times.

### Measurement of pH values after CAP treatment

The medium was stored at room temperature. Three millimetres of medium per well (six-well plates) was CAP-treated for different time doses (30, 60, 90, 120 and 150 s) without adding virus. A distance of 15 mm to the medium surface was maintained. Immediately after treatment and during the experiment, samples were kept at 37 °C and 5 % CO_2_. Untreated medium was used as a control. After calibration, pH values were measured using a pH-Meter (inoLab pH 720, Xylem Analytics Germany Sales GmbH and Co. KG, WTW, Weilheim, Germany). pH values were measured 5, 15, 30, 60, 120 and 180 min and 24 h after treatment.

### Temperature measurement after CAP treatment

The medium was stored at room temperature and treated with CAP for 30, 60, 90, 120 and 150 s. A digital temperature measurement was performed 30, 60, 90, 120 and 150 s post-treatment.

### Real-time PCR analyses

To detect HSV-1 genomes 3 h post-infection we performed real-time PCR analyses targeting the *gpD* gene in the HSV-1 genome [33]. To perform the PCR reaction we used the CFX96 Touch Real-Time PCR Detection System (Bio-Rad), and the following primers: Forw 5′-GGT CTC TTT TGT GTG GTG C-3′ and Rev 5′-GCC CAC TAT GAC GAC AAA C [33]. The PCR product results in an 84 bp amplicon. We applied the my-Budget 5x EvaGreen QPCR-Mix II reagent (Bio-Budget) using the following PCR programme: preincubation/activation at 95 ˚C for 5 min, amplification and data collection for 40 cycles (95 ˚C for 15 s and 60 ˚C for 30 s).

### Statistical analyses

All data are reported as mean+/−standard deviation. We checked the normal distribution with the Shapiro–Wilk test. All results appeared to be distributed normally (alpha=0.05). We determined the significance using the one-way analysis of variance (ANOVA) test (alpha=0.05) referring to the positive control. We visualized all QQ plots, which are displayed in Fig. S1 (available in the online version of this article).

## Results

### Optimization of infection conditions and the applied aciclovir concentration

**Fig. 1.**
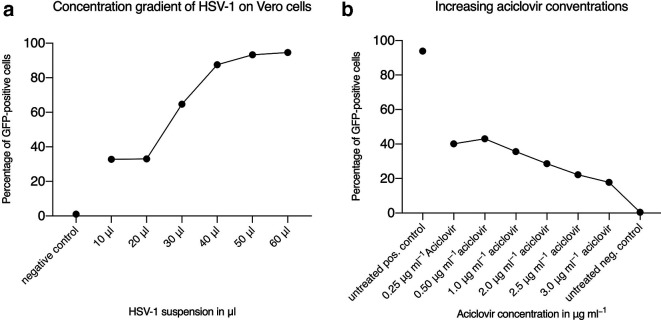
Optimization of infection conditions and the applied aciclovir concentration. The viral load was determined by measurement of GFP expression levels via flow cytometry analyses. (a) Concentration gradient to determine the optimal dose of virus resulting in high transduction efficiencies with negligible toxicity. (b) Aciclovir, as an established antiviral agent, was used as a positive control. Untreated cells represented negative controls. The mean of an experiment, which was performed in triplicate, is shown.

To identify the optimal dose for HSV-1 infection, which allows the reduction of virus titres after CAP treatment to be measured, we initially performed concentration gradients. Vero cells were infected using different dosages of HSV-1 and infectivity was measured by flow cytometry analyses. We found that applying 40 µl of the virus suspension results in 90 % GFP-positive cells and consequently used this virus load for the following experiments (Fig. 1a). Next, we tested which aciclovir concentration results in an optimal antiviral effect. As shown in Fig. 1b, a concentration of 2.5 and 3.0 µg ml^−1^ of aciclovir resulted in the highest reduction of HSV-1 infectivity. For the following experiments and for direct comparison with CAP-treated virus, an aciclovir concentration of 2.5 µg ml^−1^ was applied.

### Antiviral effect of CAP on HSV-1 and infectivity on Vero cells

**Fig. 2.**
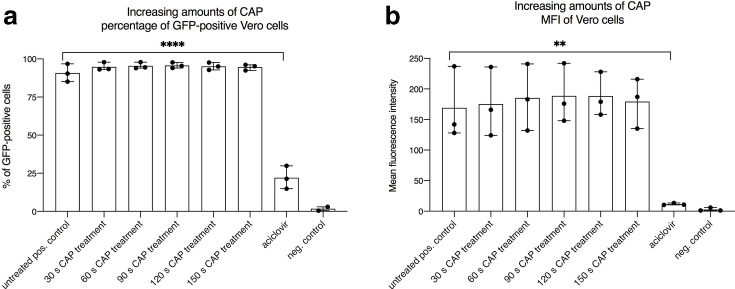
Measurements of HSV-1 infected Vero cells showed no decreased transduction efficiencies after treatment with cold atmospheric plasma (CAP). To investigate whether CAP is an effective antiviral tool, the recombinant GFP-tagged HSV-1 contained in the cell supernatant was treated with defined dosages of plasma. The kINPen med (neoplas tools GmbH, Greifswald, Germany) was used as a plasma device. (a) Percentage of GFP-positive cells measured by flow cytometry and (b) the corresponding mean fluorescence intensity (MFI). Negative control: no virus; positive control: untreated virus; aciclovir control: 2.5 µg ml^−1^. The mean+/−standard deviation is shown. Experiments were performed in triplicate and repeated three times. A statistically relevant difference (*P*<0.05) was only observed for the aciclovir groups when directly compared to the positive control.

To investigate the effect of CAP on HSV-1, 40 µl of virus suspension diluted in a total of 1 ml of medium were treated with different dosages of plasma (30s, 60s, 90s, 120s, 150s). We found that treatment with CAP had no effect on GFP expression derived from incoming HSV-1 genomes in Vero cells. There was no difference regarding the quantification of GFP-positive cells and the evaluation of the mean fluorescence intensity (Fig. 2a and b). In sharp contrast, the aciclovir control showed a significant reduction of GFP-positive cells from 95 % down to 20 % (Fig. 2a). This was in concordance with the observation that a robust decline of the mean fluorescent intensity was measured (Fig. 2b).

### Antiviral effect of CAP to HSV-1 on SH-SY5Y cells

**Fig. 3.**
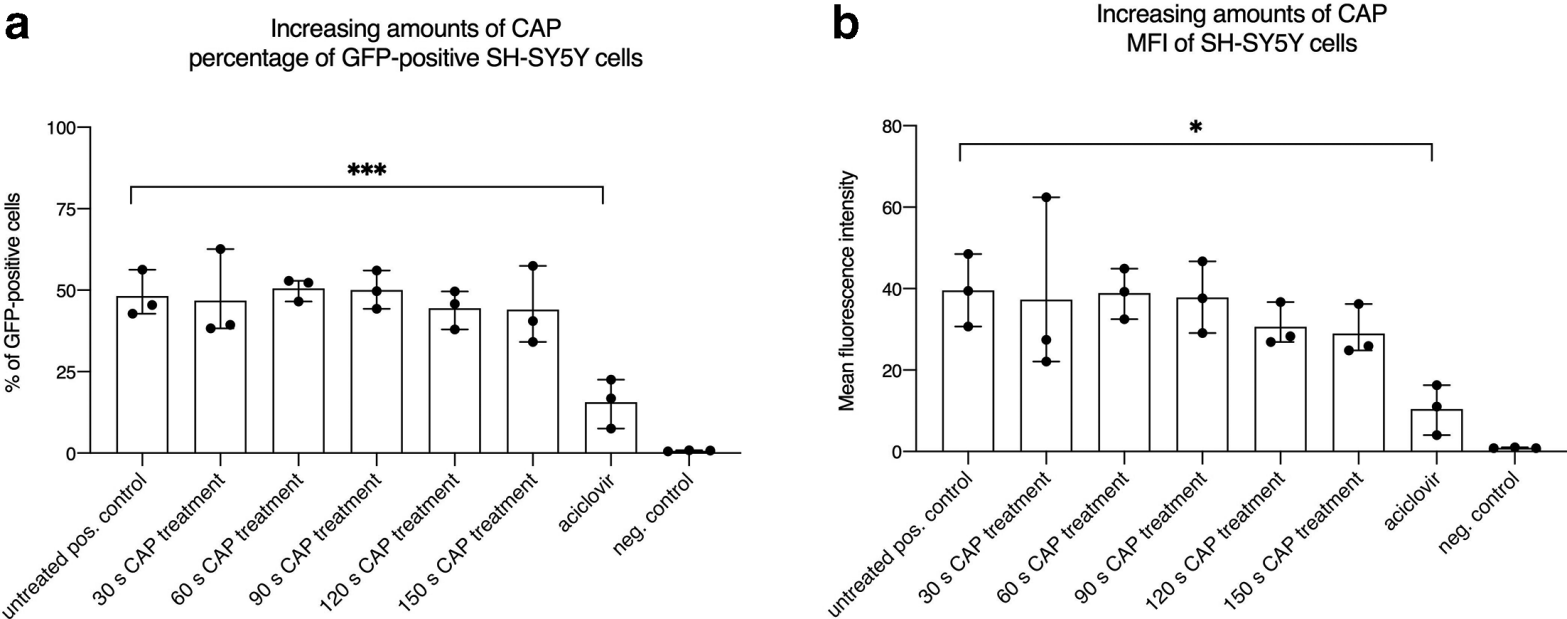
HSV-1 on SH-SY5Y cells showed no decreased transduction efficiencies after treatment with cold atmospheric plasma (CAP). (a) Percentage of GFP-positive cells measured by flow cytometry and (b) the corresponding mean fluorescence intensity (MFI). Negative control: no virus; positive control: untreated virus; aciclovir control: 2.5 µg ml^−1^. The mean+/−standard deviation is shown. Experiments were performed in triplicate and repeated three times. A statistically relevant difference (*P*<0.05) was only observed for the aciclovir groups when compared directly to the positive control.

Next, we explored SH-SY5Y cells, which were isolated from a bone marrow biopsy taken from a 4-year-old female with neuroblastoma. We used this cell line as a model for neuronal infection, which also occurs during natural HSV-1 infection. CAP treatment of HSV-1 revealed no effect on GFP-positive cells after infection of SH-SY5Y with the GFP-encoding HSV-1 virus (Fig. 3a). This was in concordance with results obtained in Vero cells. Only the aciclovir control showed a significant reduction of GFP-positive cells (Fig. 3a). With respect to the mean fluorescence intensity results, a slight but not significant reduction was visible when applying higher CAP dosages (Fig. 3b).

### Antiviral effect of CAP treatment on HSV-1 at a reduced viral dose

**Fig. 4.**
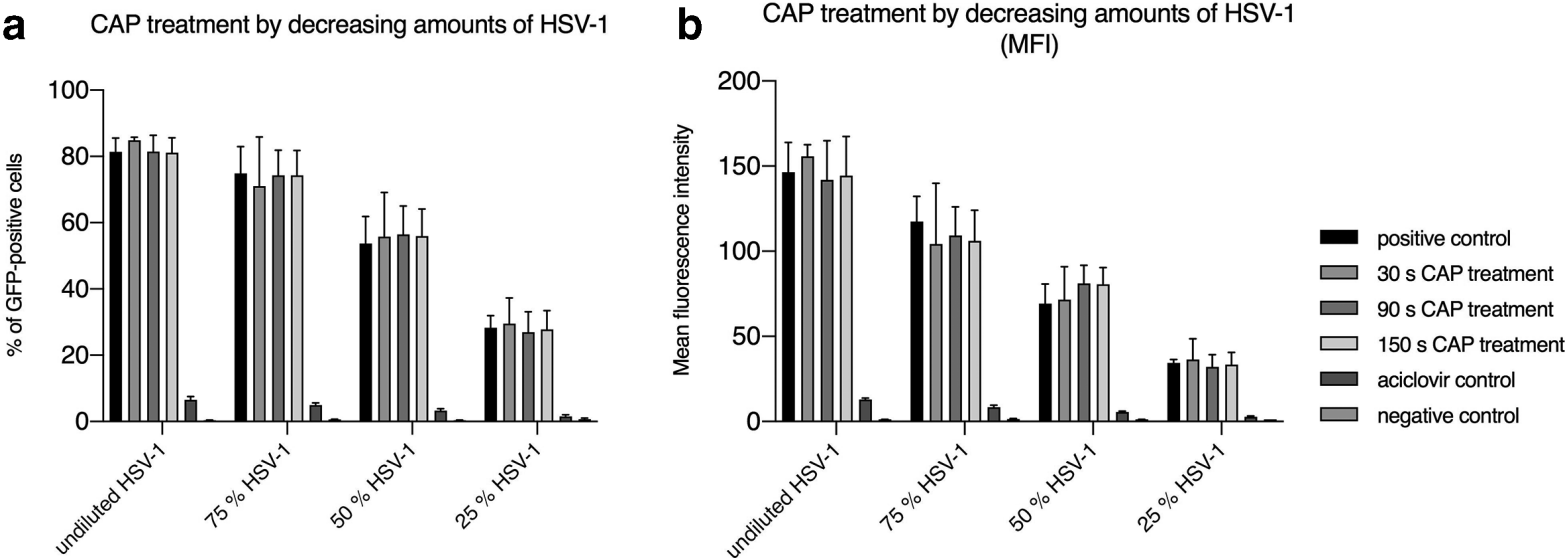
A fourfold reduction of viral load (HSV-1) on Vero cells showed no decreased transduction efficiencies after treatment with cold atmospheric plasma (CAP). (a) Percentage of GFP-positive cells measured by flow cytometry and (b) the corresponding mean fluorescence intensity (MFI). Negative control: no virus; positive control: untreated virus; aciclovir control: 2.5 µg ml^−1^. The mean+/−standard deviation is shown. Experiments were performed in triplicate and repeated three times.

**Fig. 5.**
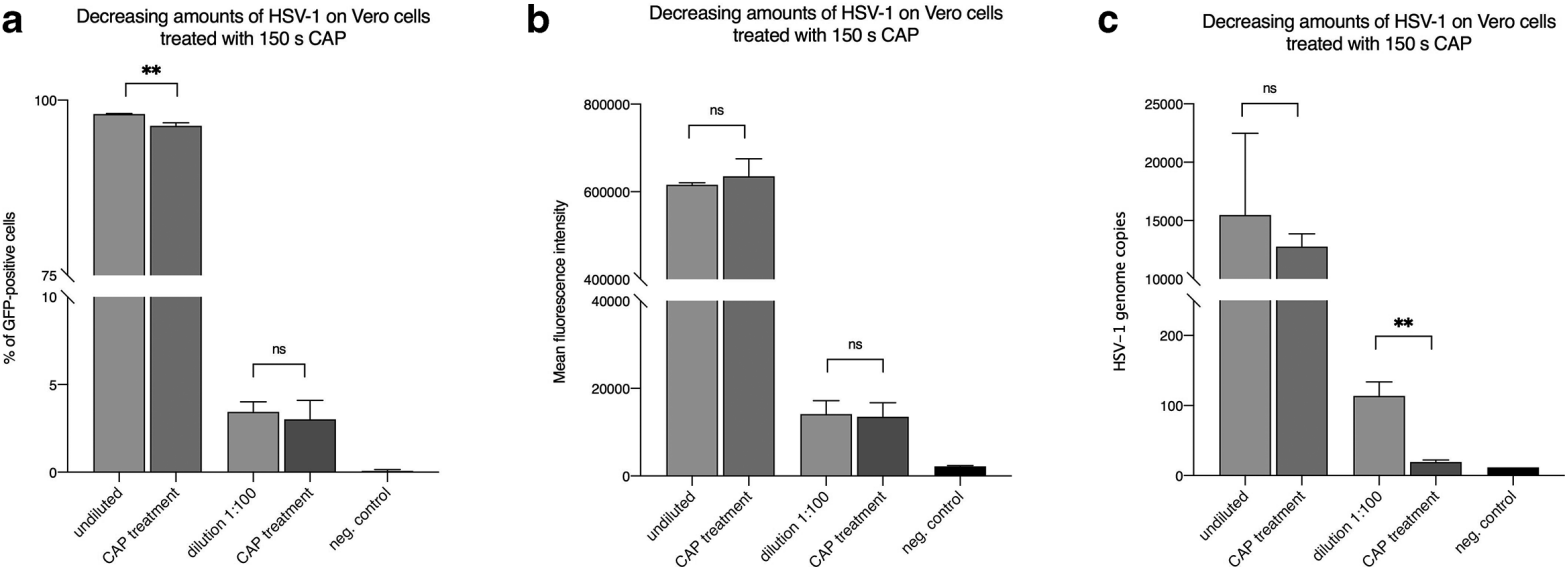
A 100-fold reduction in viral load results in reduction of HSV-1 infectivity. A 100-fold diluted virus suspension was used and HSV-1 genomes and GFP expression levels were measured 3 and 24 h post-infeciton, respectively. (a) Percentage of GFP-positive cells measured by flow cytometry and (b) the corresponding mean fluorescence intensity (MFI). (c) HSV-1 genome copy numbers were measured 3 h post-infeciton. Negative control: no virus; positive control: untreated virus. The mean+/−standard deviation is shown. Experiments were performed in triplicate and repeated three times.

We speculated that the absence of an antiviral effect of CAP on HSV-1 could be due to an excess of virus perturbing the readout of the CAP effect. We therefore reduced the virus dose in the following experiment. As shown in Fig. 4a and b a fourfold decrease in viral load did not result in a significant reduction of GFP expression. Neither the measurement of GFP-positive cells nor the analyses of the mean fluorescence intensity revealed any effect. This was in sharp contrast to the results obtained after antiviral treatment with aciclovir (Fig. 4a, b). However, when we reduced the viral load by 100-fold we observed a low but measurable effect on GFP expression levels (Fig. 5a and b). Furthermore, we measured significantly decreased numbers of HSV-1 genomes 3 h post-infection for CAP-treated viruses (Fig. 5c), which implies that CAP shows an antiviral effect at low virus dose.

## Discussion

At first sight, some results of the study presented here seem to contradict the results of the publication by Alekseev and colleagues in which an effect of CAP on HSV-1 was observed. However, looking more closely at the methods, major differences appear. In contrast to our study, only a very low viral load (m.o.i. of 0.1) was used in the cited paper [30]. Since clinical symptoms of HSV-1 such as herpes labialis, at least in the blister stage, can be assumed to involve significantly higher viral loads, we decided in this study to adapt the experimental setup by using a higher virus dose. This would more closely resemble the clinical situation. However, even after a fourfold decrease in viral load, no effect of CAP on HSV-1 replication could be observed. We then hypothesized that an even lower viral load or a longer treatment duration with CAP may lead to an effective antiviral effect against HSV-1. We confirmed this hypothesis by showing that an antiviral effect of CAP on HSV-1 is measurable after decreasing the viral load 100-fold. This antiviral effect was clearly measurable 3 h post-infection on the genome level. However, 24 h post-infection this effect was significantly less pronounced and we speculate that this may be due to robust virus replication in these 24 h, which abrogated the CAP effect. Note that a higher dose or longer treatment with CAP could have a negative effect on healthy tissue, limiting its therapeutic use, as was also observed for the inactivation of *E. coli* [34]. Therefore, it remains to be analysed whether CAP displays an antiviral effect in a clinical study.

Further differences between previous studies and the present studies are the investigated cell types and also the methods to determine the viral load after antiviral treatment. Here we explored Vero cells, which are well known in HSV-1 research. An article by Brun and colleagues briefly mentions an antiviral therapy for HSV-1 based on atmospheric-pressure cold plasma (APCP) and subsequent analyses using Vero cells [35]. The authors state that no impact on the CPE of CAP-treated HSV-1 was observed, which is in concordance with our results. Compared to the data presented here, a cell-dependent effect seems unlikely, but this cannot be ruled out completely at this time, because in total only two cell lines were analysed. The fact that the SH-SY5Y cells also showed no noticeable effect is in conflict with this speculation.

In concordance with the present study, in the studies by Brun *et al*. and Alekseev *et al*., herpesviruses were also produced in cell culture. However, the HSV-1 strains used were different. Brun *et al*. analysed strain 16 [35], Alekseev *et al*. used the KOS strain [30] and our study applied strain 17. It is known that viruses produced in cell culture are prone to mutations that do not occur *in vivo*. One example is the classical swine fever virus where the mutation occurs during cell culture [36]. In the literature, DNA viruses such as HSV-1 are described as being comparatively resistant to mutations. However, although the mutation rates of DNA viruses were reported to be low [17], only limited information is available with respect to differences in mutations in DNA viruses, which are directly derived from the patient or viruses that were amplified and serially passaged in cell culture.

It would also be conceivable that the antiviral effect of CAP varies depending on the plasma source. Three types of plasma sources may be applied under these conditions, namely barrier discharges, corona discharges and plasma jets [37]. The kINPen med, which has been used in this study, is classified under the plasma jets. In the study of Alekeseev *et al*., a dielectric barrier discharge plasma was used [30]. Moreover, another production method, such as applying another gas or plasma engine, can lead to different compositions of the reactive species and the single components of the effluent. Potentially, using a dielectric barrier discharge plasma instead of plasma jet types may lead to other effects, which may also include the temperature and the pH of the treated sample. However, as shown in Fig. S1 and in concordance with our previous study [31], the temperature and pH of the virus suspension were not changed after plasma treatment.

In conclusion, an antiviral effect of CAP on HSV-1 could only be observed at low viral dose. Further investigations with other sources of CAP and especially with other viruses should be conducted. Since little is known about the mechanism of the CAP-mediated antiviral action, and since the results of the limited amount of available studies are inconsistent, further studies should be performed. Further, a larger number of other viruses should be investigated.

## Supplementary Data

Supplementary material 1

## References

[1] Whitley RJ, Kimberlin DW, Roizman B (1998). Herpes simplex viruses. Clin Infect Dis.

[2] Kieff E, Hoyer B, Bachenheimer S, Roizman B (1972). Genetic relatedness of type 1 and type 2 herpes simplex viruses. J Virol.

[3] Groves MJ (2016). Genital herpes: a review. Am Fam Physician.

[4] Jackowski J, Striezel PDF, Altenburg A, Beck J, Hullmann M (2017). Diagnostik und Therapieoptionen von Aphthen und aphthoiden Läsionen Der Mund-und Rachenschleimhaut (S2k).

[5] Ashley RL, Wald A (1999). Genital herpes: review of the epidemic and potential use of type-specific serology. Clin Microbiol Rev.

[6] Knickelbein JE, Hendricks RL, Charukamnoetkanok P (2009). Management of herpes simplex virus stromal keratitis: an evidence-based review. Surv Ophthalmol.

[7] Stevens JG, Cook ML (1971). Latent herpes simplex virus in spinal ganglia of mice. Science.

[8] Brown JC (2017). Herpes simplex virus latency: the DNA repair-centered pathway. Adv Virol.

[9] Menendez CM, Carr DJJ (2017). Defining nervous system susceptibility during acute and latent herpes simplex virus-1 infection. J Neuroimmunol.

[10] Workowski KA, Bolan GA (2015). Sexually transmitted diseases treatment guidelines, 2015. MMWR Recommendations and reports: Morbidity and mortality weekly report Recommendations and reports.

[11] Bradshaw MJ, Venkatesan A (2016). Herpes simplex virus-1 encephalitis in adults: pathophysiology, diagnosis, and management. Neurotherapeutics.

[12] Roozbahani M, Hammersmith KM (2018). Management of herpes simplex virus epithelial keratitis. Curr Opin Ophthalmol.

[13] Crumpacker CS, Schnipper LE, Zaia JA, Levin MJ (1979). Growth inhibition by acycloguanosine of herpesviruses isolated from human infections. Antimicrob Agents Chemother.

[14] Elion GB, Furman PA, Fyfe JA, de Miranda P, Beauchamp L (1977). Selectivity of action of an antiherpetic agent, 9-(2-hydroxyethoxymethyl) guanine. Proc Natl Acad Sci U S A.

[15] Bergmann M, Beer R, Kofler M, Helbok R, Pfausler B (2017). Acyclovir resistance in herpes simplex virus type I encephalitis: a case report. J Neurovirol.

[16] Sauerbrei A (2017). Acyclovir resistance in herpes simplex virus type I encephalitis: a case report. J Neurovirol.

[17] Sanjuán R, Domingo-Calap P (2016). Mechanisms of viral mutation. Cell Mol Life Sci.

[18] Lee HJ, Shon CH, Kim YS, Kim S, Kim GC (2009). Degradation of adhesion molecules of G361 melanoma cells by a non-thermal atmospheric pressure microplasma. New J Phys.

[19] Isbary G, Morfill G, Schmidt HU, Georgi M, Ramrath K (2010). A first prospective randomized controlled trial to decrease bacterial load using cold atmospheric argon plasma on chronic wounds in patients. Br J Dermatol.

[20] Arndt S, Unger P, Wacker E, Shimizu T, Heinlin J (2013). Cold atmospheric plasma (CAP) changes gene expression of key molecules of the wound healing machinery and improves wound healing in vitro and in vivo. PLoS One.

[21] Heinlin J, Isbary G, Stolz W, Morfill G, Landthaler M (2011). Plasma applications in medicine with a special focus on dermatology. Journal of the European Academy of Dermatology and Venereology.

[22] Sladek REJ, Stoffels E (2005). Deactivation of *Escherichia coli* by the plasma needle. Journal of Physics D: Applied Physics.

[23] Sladek REJ, Filoche SK, Sissons CH, Stoffels E (2007). Treatment of Streptococcus mutans biofilms with a nonthermal atmospheric plasma. Lett Appl Microbiol.

[24] Venezia RA, Orrico M, Houston E, Yin S-M, Naumova YY (2008). Lethal activity of nonthermal plasma sterilization against microorganisms. Infect Control Hosp Epidemiol.

[25] Joaquin JC, Kwan C, Abramzon N, Vandervoort K, Brelles-Mariño G (2009). Is gas-discharge plasma a new solution to the old problem of biofilm inactivation?. Microbiology.

[26] Barton A, Wende K, Bundscherer L, Hasse S, Schmidt A (2013). Nonthermal plasma increases expression of wound healing related genes in a keratinocyte cell line. Plasma Med.

[27] Ahlfeld B, Li Y, Boulaaba A, Binder A, Schotte U (2015). Inactivation of a foodborne norovirus outbreak strain with nonthermal atmospheric pressure plasma. mBio.

[28] Guo L, Xu R, Gou L, Liu Z, Zhao Y (2018). Mechanism of virus inactivation by cold Atmospheric-Pressure plasma and plasma-activated water. Appl Environ Microbiol.

[29] Isbary G, Shimizu T, Zimmermann JL, Heinlin J, Al-Zaabi S (2014). Randomized placebo-controlled clinical trial showed cold atmospheric argon plasma relieved acute pain and accelerated healing in herpes zoster. Clin Plasma Med.

[30] Alekseev O, Donovan K, Limonnik V, Azizkhan-Clifford J (2014). Nonthermal dielectric barrier discharge (DBD) plasma suppresses herpes simplex virus type 1 (HSV-1) replication in corneal epithelium. Transl Vis Sci Technol.

[31] Bunz O, Mese K, Zhang W, Piwowarczyk A, Ehrhardt A (2018). Effect of cold atmospheric plasma (CAP) on human adenoviruses is adenovirus type-dependent. PLoS One.

[32] Datsenko KA, Wanner BL (2000). One-Step inactivation of chromosomal genes in *Escherichia coli* K-12 using PCR products. Proc Natl Acad Sci U S A.

[33] Hong YJ, Lim MS, Hwang SM, Kim TS, Park KU (2014). Detection of herpes simplex and varicella-zoster virus in clinical specimens by multiplex real-time PCR and melting curve analysis. Biomed Res Int.

[34] Sosnin EA, Stoffels E, Erofeev MV, Kieft IE, Kunts SE (2004). The effects of UV irradiation and gas plasma treatment on living mammalian cells and bacteria: a comparative approach. IEEE Trans Plasma Sci IEEE Nucl Plasma Sci Soc.

[35] Brun P, Brun P, Vono M, Venier P, Tarricone E (2012). Disinfection of ocular cells and tissues by atmospheric-pressure cold plasma. PLoS One.

[36] Hulst MM, van Gennip HG, Moormann RJ (2000). Passage of classical swine fever virus in cultured swine kidney cells selects virus variants that bind to heparan sulfate due to a single amino acid change in envelope protein E(rns). J Virol.

[37] Weltmann KD, Kindel E, von Woedtke T, Hähnel M, Stieber M (2010). Atmospheric-pressure plasma sources: prospective tools for plasma medicine. Pure and Applied Chemistry.

