# Supplementary material for: Cold atmospheric plasma as antiviral therapy – effect on human herpes simplex virus type 1
Source: J Gen Virol. 2020 Jan 21;101(2):208–15. doi: 10.1099/jgv.0.001382 (PMC7414428; doi:10.1099/jgv.0.001382)
Supplement: Supplementary material 1 [file jgv-101-208-s001.pdf]

Supplementary information

Cold atmospheric plasma as antiviral therapy - effect on human herpes simplex virus type 1

Oskar Bunz, Kemal Mese, Christina Funk, Maximilan Wulf, Susanne M. Bailer, Andree Piwowarczyk, Anja Ehrhardt

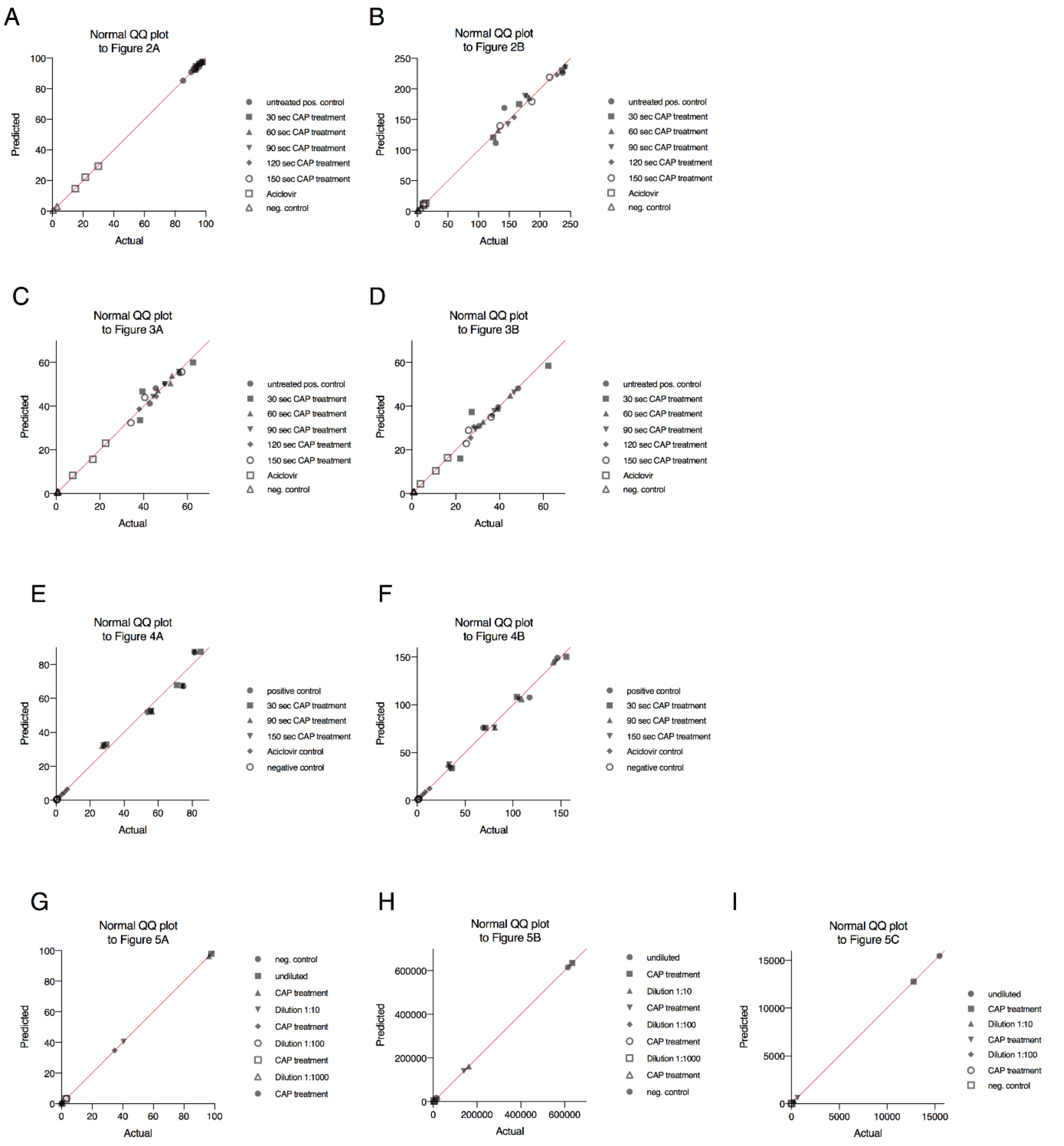

Supplementary Figure 1. Normal QQ plots of the statistical analysis of figures 2 to 5. (A-I) The QQ plots show visually that the results are normally distributed. Please not the subfigures refer to the respective main figures as mentioned in the title of the single subfigures.

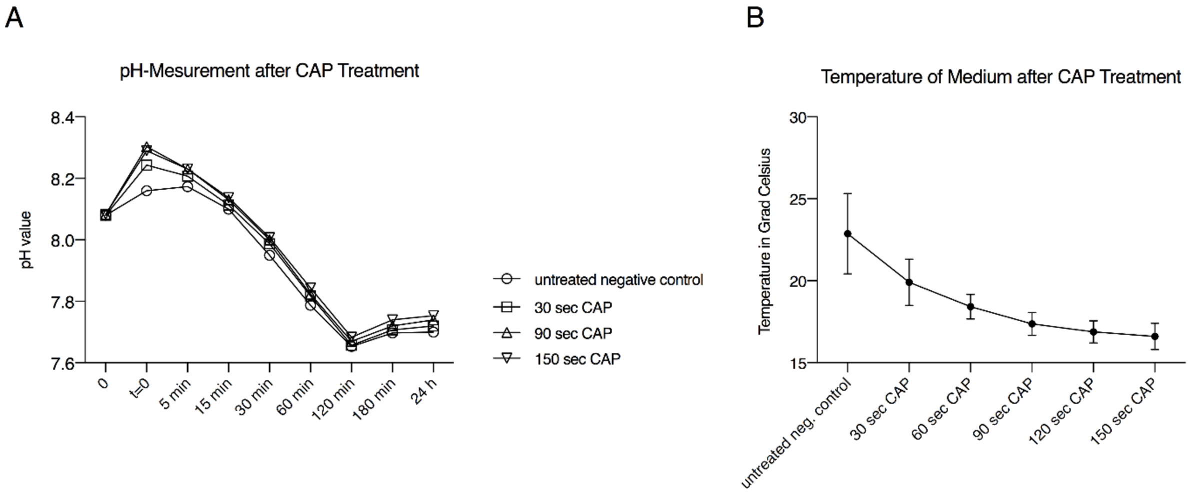

Supplementary Figure 2. Effects of CAP on temperature and pH-values of the medium. (A) Effect of different CAP dosages on the temperature of the medium. **(B)** Effect of the different dosages of CAP on the pH values of the medium used for culturing cells.
